# High-Performance Plant Pest and Disease Detection Based on Model Ensemble with Inception Module and Cluster Algorithm

**DOI:** 10.3390/plants12010200

**Published:** 2023-01-03

**Authors:** Manzhou Li, Siyu Cheng, Jingyi Cui, Changxiang Li, Zeyu Li, Chang Zhou, Chunli Lv

**Affiliations:** 1 College of Plant Protection, China Agricultural University, Beijing 100083, China; 2 College of Information and Electrical Engineering, China Agricultural University, Beijing 100083, China; 3 Institution of Big Data, China Agricultural University, Beijing 100083, China; 4 College of Economics and Management, China Agricultural University, Beijing 100083, China; 5 Yantai Institute, China Agricultural University, Yantai 264032, China

**Keywords:** plant pest and disease detection, deep learning, object detection, YOLO, Faster-RCNN, Model Ensemble

## Abstract

Protecting crop yields is the most important aspect of agricultural production, and one of the important measures in preserving yields is the control of crop pests and diseases; therefore, the identification of crop pests and diseases is of irreplaceable importance. In recent years, with the maturity of computer vision technology, more possibilities have been provided for implementing plant disease detection. However, although deep learning methods are widely used in various computer vision tasks, there are still limitations and obstacles in practical applications. Traditional deep learning-based algorithms have some drawbacks in this research area: (1) Recognition accuracy and computational speed cannot be combined. (2) Different pest and disease features interfere with each other and reduce the accuracy of pest and disease diagnosis. (3) Most of the existing researches focus on the recognition efficiency and ignore the inference efficiency, which limits the practical production application. In this study, an integrated model integrating single-stage and two-stage target detection networks is proposed. The single-stage network is based on the YOLO network, and its internal structure is optimized; the two-stage network is based on the Faster-RCNN, and the target frame size is first clustered using a clustering algorithm in the candidate frame generation stage to improve the detection of small targets. Afterwards, the two models are integrated to perform the inference task. For training, we use transfer learning to improve the model training speed. Finally, among the 37 pests and 8 diseases detected, this model achieves 85.2% mAP, which is much higher than other comparative models. After that, we optimize the model for the poor detection categories and verify the generalization performance on open source datasets. In addition, in order to quickly apply this method to real-world scenarios, we developed an application embedded in this model for the mobile platform and put the model into practical agricultural use.

## 1. Introduction

From sowing, growing to harvesting, crops are often affected by a variety of factors, including many environmental factors, such as temperature [1], moisture, soil, physical factors [2], pests, etc. Among them, pests are a very important factor, containing many kinds of pests (plant pathogens, pests, weeds and rodents, etc.), which affect the yield and quality of cultivated plants and have an impact on fossil energy reserves [3,4,5,6,7,8]. Agricultural pests are usually classified as insects and mites that cause damage to various plants, and insects are the largest number of pests that affect crops. All kinds of plant damage caused by them are called pests. After insects injured or exposed to pathogens, the organic content of the plant will change [9] causing its mutation, leading to wilting and even death [10]. In recent years, crops are always hard to escape the attack of pests.

Globally, more than 80% of agricultural output comes from farmers, and more than 50% of the output is lost due to pests and diseases, resulting in a large-scale disruption of food supply and large numbers of hungry people [11]. According to a study released by the FAO in June 2021, due to the influence of climate change, plant pests that damage important cash crops are becoming more destructive and increasingly threatening food security and the environment. The FAO estimates that the annual loss of plant diseases to the global economy exceeds 220 billion dollars. In addition, up to 40% of global crop yield is lost to pests every year, resulting in at least 70 billion dollars in losses. At the same time, it is find that climate change will increase the risk of pests transmission in agricultural and forestry ecosystems, especially in cold Arctic, northern, temperate and subtropical regions. Moreover, due to the warming of the climate, some pests, such as the meadow crab moth, are already spreading further as a result of warming. Other species such as the desert locust (the world’s most destructive migratory pest in the world) are expected to change their migratory routes and geographical distribution due to climate change. According to the results of a large-scale monitoring survey in China from 2019 to 2022, the occurrence of grasshoppers in China’s grasslands still has obvious regional features. In terms of area of occurrence, the annual breeding areas in Southwest China and South China account for more than 80% of the total area of occurrence, while the middle and lower reaches of the Yangtze River and the transitional areas of migration such as Jianghuai account for 10–20% of the total area of occurrence, and the key prevention areas in the north account for less than 1% of the total area of occurrence counties, there are 1645 counties in 2019–2020, including 1541 counties in 26 provinces are involved in 2019 and 1423 counties in 27 provinces are related in 2020, accounting for 80% of the occurrence counties in these two years. In 2021, it is expected that the meadow moths will be occur in large numbers in southwest China, South China, middle and lower reaches of Yangtze River and Jianghuai region, which need to be key prevention and control. During the period of adult moth infestation in the south, when there is a suitable East Asian monsoon or typhoon event, it can help the insects to migrate northward. Besides, Northwest China, Yellow and Huaihua, North China and Northeast China need to strengthen prevention. It is estimated that the national disaster area is more than 20 million mu, and the control area is more than 30 million mu times. Therefore, it is an important task to quickly and efficiently determine the scope of prevention. For another example, in 2021, rice planthoppers will re-occur in a large scale in the rice areas of South China, Jiangnan and the middle and lower reaches of the Yangtze River, and moderately occur in rice areas in other south. It is expected that there will be 350 million mu in the whole country, and 450 million mu will be controlled. Cnaphalocrocis medinalis, as an important agricultural pest, will have serious disasters in rice areas in the south of the Yangtze River, the eastern of southwest China, and the lower reaches of the Yangtze River in 2021, and moderate disasters in south China, the western of southwest China and Jianghuai. It is expected to occur in an area of 210 million mu nationwide and to be controlled in an area of 250 million mu. It can be seen that the pests are characterized by rapid damage, heavy losses and great difficulties in prevention and control, with a wide range of pest species, different forms and different patterns of occurrence. Agricultural pests mainly affect rice, corn, wheat [12], potatoes, soybeans, sunflowers, vegetables, fruit trees, etc. They are important food crops and food for people’s living standards in China [13], and some of them also play an important roles in industrial manufacturing and medical applications, Therefore, the control of agricultural pests is of great importance [14].

IIt is an important subject to study the effects of different degrees of disease on crop yield loss, so as to formulate reasonable control indicators and measures and obtain maximum economic benefits [15,16,17,18]. The yield of crops is the result of many factors, so it is a very complicated problem to estimate the degree of yield loss caused by diseases. Most foliar pests and diseases in crops are caused by pathogens that suck nutrients from the host, reduce the photosynthetic leaf area and interfere with the accumulation of organic matter and water physiology, but do not directly affect the harvested parts of the crop, such as fruits and ears [19,20,21]. Therefore, the relationship between disease severity and yield is more complicated, and it is more difficult to determine the disease types according to the symptoms and signs of leaves. Thus, it is important to study the effects of various pests and diseases on crop yield loss, and to formulate reasonable control indicators and measures more easily and quickly. In the practice of pest control, first of all, it is necessary to correctly identify beneficial insects and pests, and to be able to make good use of beneficial insects and control pests [22]. Secondly, we need to master the general morphological characteristics of insects and their growth and development rules, and find out the weaknesses of insect life to control them, so as to achieve get twice the result with half the effort. Moreover, pests have different morphological characteristics in different growth periods, so they should be accurately identified in different periods, such as eggs, nymphs, larvae, adults, etc.

Currently, pest identification in agricultural production relies mainly on visual and empirical judgments by farmers or remote expert consultations [14]. However, these methods have obvious shortcomings, such as low recognition accuracy and long cycle time. Low recognition accuracy may directly lead to wrong control methods, which may produce the opposite effect, while long cycle time may miss the best control period and lead to economic losses. With the concept of precision agriculture and the application of computers in agriculture, machine vision and deep learning are put forward. When recognizing and detecting plant diseases and pests, the deep learning and convolutional neural net works (CNN) have quickly become the preferred methods. Compared to the previous detection methods which relied on the experience of farmers, this method is not only more accurate, but also time efficient. CNN is suitable for detecting diseases since it has the ability to learn the image features, such as pattern, color and texture. And deep learning technology made it possible to accurately analyze disease and pest species on the plant [23]. It can effectively help farmers improve crop quality and reduce economic losses of agricultural production. After finding out the disease, the appropriate pesticides or various methods can be used to shelter from the rain and wind [24].

Although deep learning methods are widely used in various computer vision tasks, there are still limitations and obstacles in practical applications.

1. In order to increase the recognition accuracy, the computational complexity needs to be increased, which inevitably leads to the decline in speed of detection.
2. Most existing studies focus on recognition efficiency at the expense of reasoning efficiency, so the utility of these models in practice is unsatisfactory.
3. Data amplification is a key component of training deep learning models. Highly accurate models require a lot of data, and the disease data for many plants are still in a blank stage. The diversity of the datasets is limited.
4. Due to mutual interference of features between different kinds of lesion spots. Multiple diseases in a plant image may reduce the accuracy of disease diagnosis.

Driven by these deficiencies, the main novelty of this work is:

1. In this paper, a high resolution dataset containing 37 pests and 8 diseases with 18,907 images was collected and produced.
2. By adding the Inception module to the YOLOv3 model and using four different sizes of convolutional kernels (1×1, 3×3, 5×5, 7×7) to perform multi-scale feature extraction and fusion in parallel, this paper proposes the inc-YOLO model.
3. by clustering the anchor box and increasing the anchor box types, this paper proposes the cluster-RCNN model, and achieves a better localization effect.
4. By integrating the above two models, our method finally achieves 85.4% accuracy in the detection of 37 pests and diseases. This method provides a feasible solution to achieve fast and efficient pest detection.
5. In this paper, a mobile application is created based on the proposed method, will be discussed in Section 6.3.

The rest of this paper is divided into six parts: the Section 2 describes the recent progress of the target issue in this paper; the Section 3 and Section 5 introduce the dataset and design details of the our model; the Section 4 shows the experimental results as well as their analysis; the Section 6 conducts numerous experiments to verify the effectiveness of the optimized method and the limitation of our methods; and the Section 7 summarizes the paper.

## 2. Related Work

**Figure 1:**
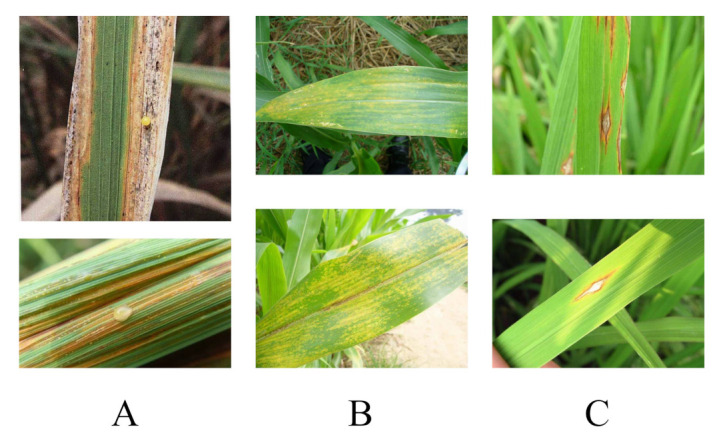
The three main diseases mentioned in [30]. (**A**) is Bacterial Leaf Blight. It is a disease that occurs in rice and is caused by the bacterium Leucaena leucocephala. The disease affects leaves and can also infect leaf sheaths. It is a phytosanitary target in China. (**B**) is Brown Spot.It is a fungal disease caused mainly by the fungus Bacillus subtilis. (**C**) is Leaf Blast. Rice blast occurs throughout the rice reproductive period and can be classified into seedling blast, leaf blast, node blast, spike blast and grain blast depending on the period and location of damage. Leaf plague can occur throughout the entire reproductive period, and the damage is heavier from tillering to nodulation. Leaf plague directly affects the normal growth of rice, and in severe cases, the plant will die, leading to a reduction in rice production or even a crop failure.

Computer vision technology has an important role to play in various industries, and plant pest identification is no exception [25,26]. Bedi, P. and Gole, P. et al. proposed a novel hybrid model of CAE and CNN for plant disease detection, which can be performed automatically. This is the first time that a hybrid system based on CAE and CNN is used for automatic detection of plant diseases. This model is mainly used to detect bacterial spot disease on leaves of peach trees [3]. owmyalakshmi, R. et al. proposed ResNset v2 model and best weighted extreme learning machine (CNNIR-OWELM for deep convolutional networks for accurate detection of initial rice plant diseases in a smart agriculture environment [27]. Rashwan, S. A. et al. improved the detection of plant leaf diseases using deep convolutional neural network (DCNN) model-driven computer vision algorithms. By using MobileNetV2, a DCNN model for embedded devices, and AlexNet, a heavy DCNN model designed for non-embedded devices, for detection, the accuracy of detection was effectively improved [28]. Joshi, K. et al. on the other hand, used digital image processing and machine learning algorithms to improve the process of disease detection on various leaves. It has the advantage of requiring less time compared to other deep learning based methods and is widely used to detect various plant and leaf diseases [29]. Latif et al. proposed a method based on deep convolutional neural network (DCNN) transfer learning for accurate detection and classification of rice leaf diseases, which also includes a VGG19 based transfer learning method. The proposed modified system can accurately detect and diagnose six different categories: healthy, narrow brown spot, leaf scald, leaf blight, brown spot and bacterial leaf blight, achieving the highest average accuracy of 96.08%. The corresponding precision, recall, specificity and F1 scores were 0.9620, 0.9617, 0.9921 and 0.9616 [30], respectively. As shown in Figure 1, the common rice foliar pests listed by this team are the main subjects of their experiments.

Sharma, A. et al. Plant leaf disease detection using transfer learning was improved in JPEG compressed domain. To improve the classification efficiency, the JPEG compressed stream consisting of DCT coefficients is fed directly into the neural network [31]. Mohameth, F. et al. combined smartphone and computer vision to make disease diagnosis through smartphone assistance a reality. By training several deep learning architecture models, the performance of some models reached more than 99.53%. At the same time, migration learning and deep feature extraction were applied to evaluate the CNN architecture and all the obtained features were also classified by SVM and KNN, and the results showed that SVM is an excellent classifier for leaf disease detection [32]. Hussain, S. A. et al. used image processing to extract features from images of plant leaves, classify the images by graphical user interface (GUI) to determine the detected diseases and calculate the affected areas, show the percentage of disease detection, using algorithms of K-means clustering and support vector machines, for comparing and detecting the percentage of diseases. The results were analyzed by mean, entropy, variance, kurtosis, skewness, contrast, and homogeneity. This model monitors the status and updates the information in the Internet of Things (IOT), thus enabling effective management of plant disease detection [33]. Kiratiratanapruk, Kantip et al. studied six varieties of major rice diseases rice blast, bacterial leaf blight, brown spot, narrow brown spot, bacterial leaf stripe and rice dwarf virus disease. Preprocessing models such as Faster R-CNN, RetinaNet, YOLOv3 and Mask RCNN were used and their detection performance was compared. The experimental results showed that YOLOv3 provided the best performance in detecting and classifying rice leaf diseases with an average precision (mAP) of 79.19%. The accuracy of Mask R-CNN, Faster R-CNN and RetinaNet was mixed with 75.92%, 70.96%, 36.11% [34]. Pan, S. Q. et al. pre-trained the model Google Net based on DCNN, and develop loss functions for deep facial recognition tasks, such as Arc Face, Cos Face and A-Softmax, were applied to detect NCLB. The results obtained that the pre-trained Google Net architecture with Softmax loss function can achieve excellent accuracy of 99.94% on NCLB diagnosis. The analysis was implemented in Python through two deep learning frameworks Pytorch and Keras. They explored the intelligent recognition techniques for NCLB and effectively diagnosed NCLB [35] from images of corn. Ssh, A et al. used diseased tomato leaf samples for their study. Firstly, histogram equalization was used to improve the quality of tomato samples, then K-means clustering was introduced to divide the data space into Voronoi cells, then contour tracing was used to extract edges, and then support vector machine (SVM), convolutional neural network (CNN), and K-nearest neighbor (K-NN) were used to classify the features. The accuracy of SVM is 88%, K-NN is 97%, and CNN is 99.6% [36], obviously, K-NN works best, CNN is similar to it, and SVM has a big difference with the first two.

In other aspects of pest identification, there are also many discoveries and advances. Oppenheim, D. et al. were the first to pioneer the implementation of deep convolutional networks for disease identification in potato tubers. The model was trained in different training-test partitions, tested on image datasets taken with standard low-cost RGB (red, green, blue) sensors, and showed high accuracy [37]. Ramaprasad, R. et al. firstly proposed a new set of baselines for classifying images into diseases. Secondly, a stacked combination for multiple disease classification (SEMFD-Net) was proposed, which is an ensemble model by stacking baseline models and using feedforward neural networks as meta-learner, with significantly optimized performance, better than the original level [38]. Detection of plant diseases with the help of threshold segmentation and random forest classification in the investigation by Kailasam, Swathi et al. This work developed a different approach for early stage crops and implemented a new disease finding system with 97.8% recognition accuracy and 99.3% true optimism and achieved a peak signal to noise ratio (PSNR) of 59.823, a structural similarity index measure (SSIM) of 0.99894, a machine squared error (MSE) value of 0.00812, with very good results and achieved some degree of innovation and improvement [39]. An automated system for plant disease detection using machine learning methods has been proposed by previous authors. Since most of the existing ML techniques for plant disease identification are based on handcrafted features, they rarely deal with large amounts of input data, and in which AlexNet and VGG19 CNN are the basis for pre-training, it is possible to obtain feature extraction for a given data with fine-tuned details. After convolutional neural network feature extraction, it selects the best subset of features by correlation coefficients and feeds them into classifiers, including K-nearest neighbors, support vector machines, probabilistic neural networks, fuzzy logic and artificial neural networks. The method proposed by Muhammad et al. was improved by augmentation steps and achieved an average accuracy of over 96% on their own collected dataset [40]. Sharma, P. et al. built a new model that more than doubled the performance of the S-CNN model trained using segmented images compared to the F-CNN model trained using complete images, and achieved an accuracy of 98.6% with 10 disease classes. Meanwhile, they verified that the confidence level of self-classification of the S-CNN model is a significant improvement over the F-CNN model by using tomato plants and target spot disease types as examples [41]. Roy, A. M. et al. proposed a high-performance real-time fine-grained object detection framework that addresses several common problems in plant pest and disease monitoring such as dense distribution, irregular morphology, multi-scale object classes, and texture similarity. The model is an improved version of the You Only Look Once (YOLOv4) algorithm, with a modified network structure incorporating DenseNet optimized feature transfer and reuse in the backbone, and two new residual blocks in the backbone and neck to enhance feature extraction and reduce computational cost. The Spatial Pyramid Pool (SPP) enhances the perceptual field, and the modified Path Aggregation Network (PANet) preserves fine-grained local information and improves feature fusion. The Hard-Swish function is also used as the primary activation, which effectively improves the accuracy of the model. At a detection rate of 70.19 FPS, the proposed model obtained an accuracy of 90.33%, an F1 score of 93.64%, and a mean average precision (mAP) value of 96.29% [42].

## 3. Materials

### 3.1. Dataset Analysis

The images were collected from the Science Park of the West Campus of China Agricultural University and the Bayannur Botanical Garden of Inner Mongolia Autonomous Region, China, and included images of foliage with pests and diseases grown under natural conditions, as well as images from the Internet. The image acquisition equipment was Canon 5D, and the resolution was 4096×2160, and the acquisition time was from 2020.2 to 2022.3. The resolution we use in training the model varies depending on the model, as shown in Table 1.

**Figure 2:**
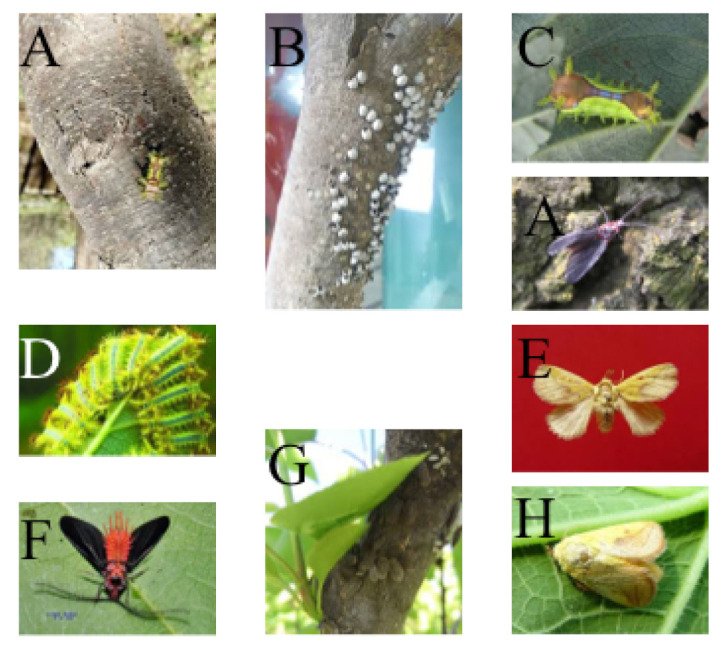
Illustration of different species of pest data sets. (**A**,**C**) are *Cnidocampaflavescens* (Walker, larva). (**F**,**I**) are *Cnidocampaflavescens* (Walker, adult). (**B**) is *Erthesinafullo* (Thunberg, egg). (**D**,**G**) are *Drosicha corpulenta*(Kuwana, adult). (**H**) is *Drosicha corpulenta* (Kuwana, larva). (**E**) is *Parasa consocia* (larva).

There are 37 pest and disease categories: *Anoplophora chinensis* (adult), *Micromelalopha troglodyta Graeser* (adult), *Apriona germari Hope* (larva), *Erthesinafullo* (Thunberg, larva), *Sericinus montelus Gray* (egg),*Cnidocampaflavescens* (Walker, cocoon), *Erthesinafullo* (Thunberg, adult), *Clostera anachoreta* (larva), *Erthesinafullo* (Thunberg, larva), *Sericinus montelus Gray* (larva), *Hyphantria cunea* (pupa), *Apriona germari Hope* (adult), *Psilogramma menephron* (pupa), *Plagiodera versicolora* (larva), *Sericinus montelus Gray* (adult), *Plagiodera versicolora* (egg), *Anoplophora chinensis* (larva), *Parasa consocia* (larva), *Cnidocampa flavescens*(Walker, adult), *Clostera anachoreta* (egg), *Plagiodera versicolora* (adult), *Hyphantria cunea* (larva), *Cnidocampa flavescens* (Walker, larva), *Psilogramma menephron* (larva), *Monochamus alternatus* (adult), *Hyphantria cunea* (adult), *Drosicha corpulenta* (Kuwana, adult), *Clostera anachoreta* (adult), *Psilogramma menephron* (adult), *Drosicha corpulenta* (Kuwana, larva), *Parasa consocia* (adult), *Spilarctia subcarnea* (Walker, adult), *Micromelalopha troglodyta* (Graeser, larva), *Spilarctia subcarnea* (Walker, larva), *Hyphantria cunea* (egg), *Erthesina fullo* (Thunberg, egg), *Monochamus alternatus* (larva). Distribution and presentation of pest datasets are shown in Table 2 and Figure 2.

**Figure 3:**
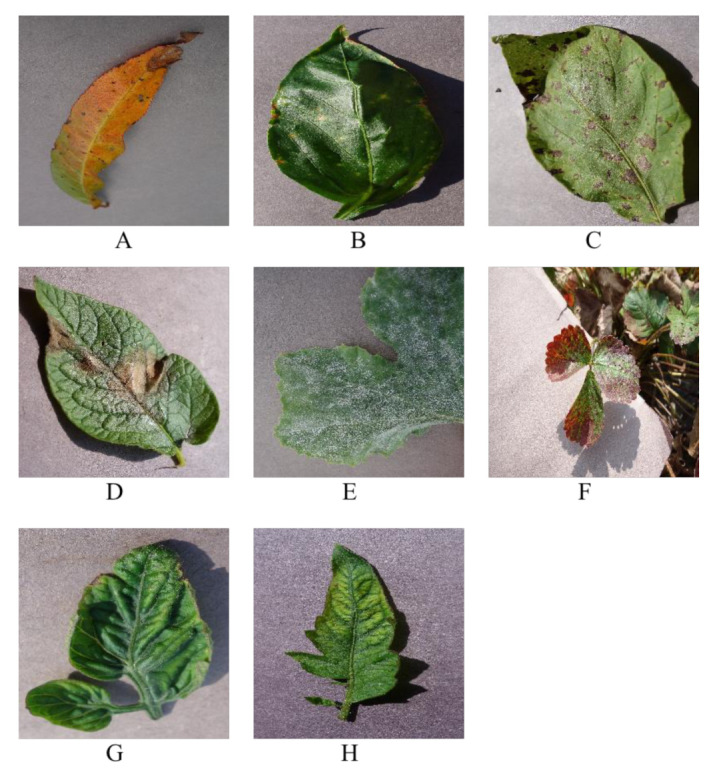
Illustration of disease data sets. (**A**) is peach bacterial spot, (**B**) is pepper bacterial spot, (**C**) is potato early blight, (**D**) is potato late blight, (**E**) is squash powdery mildew, (**F**) is strawberry leaf scorch, (**G**) is tomato curl virus, (**H**) is tomato mosaic virus.

The disease dataset used in this paper includes images collected from the West Campus of China Agricultural University, Haidian District, Beijing, China, and from Wayi Community, Sudulun Town, Ullat Qianqi, Bayannur City, Inner Mongolia, and some web images. It contains 6 species with 8 diseases, such as peach bacterial spot, pepper bacterial spot, potato early blight, potato late blight, squash powdery mildew, strawberry leaf scorch, tomato curl virus, tomato mosaic virus.The dataset contains 7199 images, as shown in Table 3 and Figure 3.

From Table 2 and Table 3, we can see that the dataset used in this paper is unbalanced, with *Psilogramma menephron* (larva) and peach bacterial spot being very low in the pest and disease datasets, which causes the model to fail to learn the features of these subclasses effectively. Because of their low frequency, the loss function cannot be effectively penalized. Therefore, this dataset is preprocessed in this paper.

### 3.2. Dataset Pro-Processing

In order to improve the detection accuracy of the model for different lighting, devices and environments, various pre-processing operations are performed on the dataset. The ImageNet dataset is also used to help the model training by migration learning. In this paper, the dataset is divided in the ratio of 7:3, in which 70% of the data are used to train the model and 30% are used for model testing.

#### 3.2.1. Overlapping Optimization

**Figure 4:**
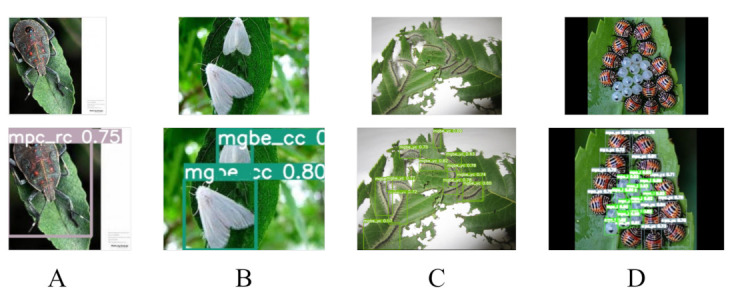
Detection results for different density scenarios on our method. (**A**–**D**) indicates the objects to be recognized from sparse to dense.

In order to improve the accuracy of detection by allowing the model to learn more detailed features with overlapping, the dataset is classified and labeled as follows:

1. No overlapping, with complete features, as shown in Figure 4A;
2. Objects are dense, and there is overlapping, as shown in Figure 4C,D;

By labeling the data sets with overlapping, the model learns the overlapped features and effectively improves the detection accuracy of images with a large number of overlapped cases without affecting the detection of normal data sets thus achieving better disease detection results.

#### 3.2.2. Dataset Enhancement

The recognition of pests and diseases is highly dependent on the shooting environment, such as lighting and angle. Image quality affects recognition accuracy, and in order to make the model have stronger generalization ability, data enhancement techniques are used to perform operations such as random brightness increase and decrease, random rotation and mirror flip on existing images. When randomly increasing or decreasing the luminosity, the Equation is followed:

(1) Output=α×Input+β

**Figure 5:**
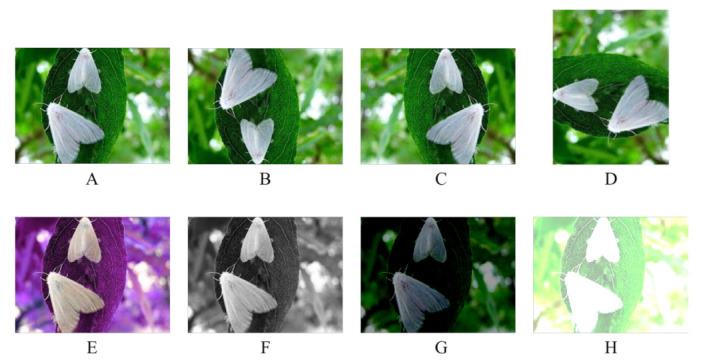
Illustration of different enhancement methods. (**A**) is the original image, (**B**) is vertical flipping, (**C**) is horizontal flipping, (**D**) is 90 degree rotation, (**E**) is hue adjustment, (**F**) is saturation adjustment, (**G**,**H**) are brightness adjustment.

In Equation (1) α stands for contrast, β stands for brightness. After the image has been randomly photoluminance incremented or decremented, the image is normalized to between [−1,1], followed by a random rotation of the rotation center point by a certain angle, and then a mirror flip. All these enhancement operations are performed automatically during training and the effect of various enhancement methods is shown in Figure 5.

## 4. Results

In this section, the model introduced in Section 5 was implemented for object detection. We trained the datasets with three input sizes, 300 × 300, 416 × 416, and 608 × 608, which are the suggested input resolutions for the models.

### 4.1. Validation Results

MobileNet’s mAP, the pre-training parameters obtained by employing COCO and PVD, is 56.7%, which is the worst performance among all models. But it is the fastest among the seven models with FPS of 34%, although it is not the lowest resolution. The speed of SSD and RefineDet, which are the lowest resolution, are in the middle level, with 24% and 15% respectively, between which SSD is about 1.6 times faster than RefineDet. Meanwhile, SSD’s *P*, *R* and mAP are 63.8%, 59.3% and 58.7% respectively, while RefineDet’s *P*, *R* and mAP are 67.8%, 62.1% and 65.9% respectively, all in the middle level among the seven models.

YOLO v5’s *P*, *R* and mAP are the best in the YOLO series, i.e., YOLOv3 and YOLOv5 comparisons, reaching 81.0%, 78.6%, 80.7% to be exact. At the same time, this performance exceeds any of the other seven comparable models with a significant advantage, especially compared to MobileNet with a difference of about 23–25%, which is a significant enhancement. The model most similar to YOLO v5 is EfficientDet with 72.1%, 69.2% and 69.7% for *P*, *R* and mAP, respectively. However, its inference speed is 7%, which is significantly lower than that of YOLO v5, reaching only 21% of the latter. This may be due to the stronger performance of the attention extraction module in YOLO v5. The same speed of 7% as EfficientDet is also Faster-RCNN, whose P,R and mAP are 60.7%, 59.3% and 60.3% respectively, and its performance is only superior to MobileNet among the seven models, but the difference in speed is larger.

For the parameters in the backbone of the model in this paper, we chose pre-training parameters obtained based on ImageNet. In addition, its Presicion, Recall and mAP outperform other comparable models, with the highest being the ensembled model with 85.2%, 84.8% and 85.0% for *P*, *R* and mAP, respectively. However, our model has no advantage in inference speed (the best Inc-YOLO and YOLO v5 in terms of speed only reach 79% of YOLO v5 at the same resolution), which may be due to the parallel network in the inception structure. Cluster-RCNN has an intermediate performance among our three models, but is the slowest, which is due to its two-stage network structure and clustering algorithm.

### 4.2. Detection Results

**Figure 6:**
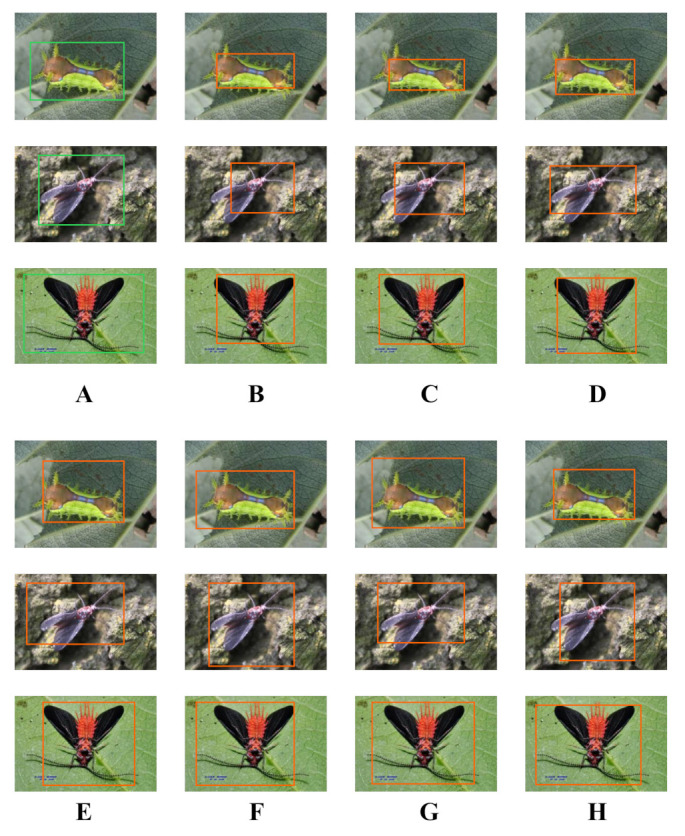
The detection results of other models. (**A**) is the groundtruth; (**B**) is the MobileNet; (**C**) is the Faster-RCNN; (**D**) is the SSD; (**E**) is the RefineDet; (**F**) is the EfficientDet; (**G**) is the YOLOv3; (**H**) is the YOLOv5.

**Figure 7:**
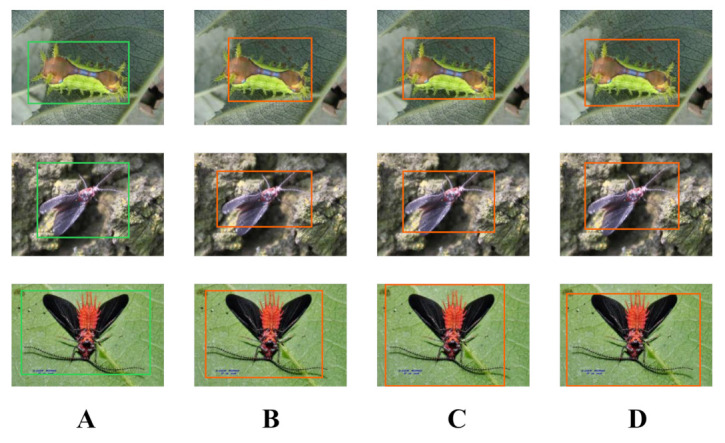
The detection results of our model. (**A**) is the groundtruth; (**B**) is the inc-YOLO; (**C**) is the cluster-RCNN; (**D**) is the ensembled model.

As shown in the Figure 3 in Section 3.1, we focus on the results of pest detection in this section since the disease images only need to perform the classification task and not the detection task. For further comparison, we extracted some images from the test set. The reason for using these images for this presentation is that these images show as many detection scenarios as possible in the dataset. Figure 6 and Figure 7 depict the detection results. The green boxes denotes the ground truth and the red boxes denote the predicted bounding boxes.

It can be witnessed that SSD performs very poorly in these images, while EfficientDet and YOLO series perform relatively well and detect lesions accurately. However, when the detected objects are too tiny, all models’ performance decreases, and part of models even have some unlabeled detected objects. This situation is probably related to the attention extraction module in these networks.

As described in Section 3.2.1, in a real scene, the performance of the model may be affected when there are multiple objects to be recognized in a single image. Therefore, in this section, we focus on the recognition effect of the model in different density scenes. The recognition results are shown in Figure 4.

From Figure 4, we can see that our model performs well in different density scenarios, even when detecting moderately dense objects. Although there is still room for improvement, it has outperformed other models. On the one hand, we augment the image before it is fed into the backbone. On the other hand, before generating the anchors, we use clustering methods to cluster them so that the generated anchor size matches the target size as much as possible.

### 4.3. Test on Other Dataset

In order to investigate whether the proposed method is widely applicable, in this subsection, we use the open source dataset proposed by [47] to test and compare the results with the model proposed by [47], as shown in Table 4.

## 5. Methods

In order to balance performance and speed, this paper proposes an integration scheme that integrates two target detection networks in single and double phases.

### 5.1. Inc-YOLO

#### 5.1.1. YOLO v3

YOLOv3 [48], as a representative algorithm of one-stage target detection algorithm, performs feature extraction on the detection image and predicts target bounding box and target class probability directly from the detection image, which has obvious speed advantage and can quickly perform disease detection. YOLOv3 uses Darknet-53 constructed by residual network as a feature extraction network, and performs high and low layer feature fusion by convolution and upsampling operations, and performs feature fusion and independent prediction on three feature maps thus improving detection accuracy. However, the deepening of the number of layers of the feature network makes it difficult to extract feature information from small-sized targets, and also lacks diverse sensory fields for each size of the feature map. Therefore, the speed advantage also leads to its weakness in detecting small-sized and easily clustered objects, which is especially obvious in target detection, similar target detection, and occlusion situation detection.

#### 5.1.2. Inception Model

**Figure 8:**
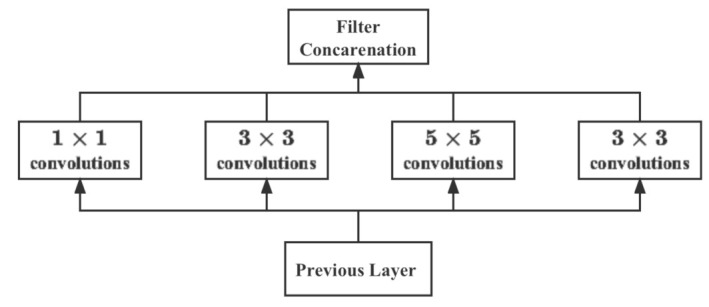
Original inception module.

In contrast, GoogLeNet [49] reduces the computational parameters of the network model by increasing the network width and using multiple Inception modules to obtain good feature extraction results. The Inception module shown in Figure 8 has multiple branches of convolution operation, which performs multiple convolution or pooling operations on the input image at different scales in parallel, and fuses the results of all branches to improve the scalability of the model while extracting both sparse and dense features of the image to achieve better results.

#### 5.1.3. Proposed Model

**Figure 9:**
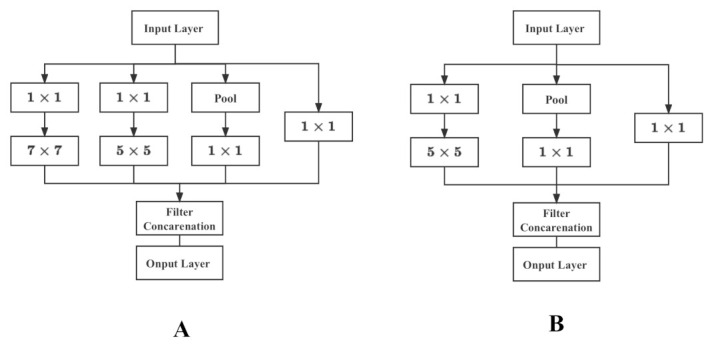
Modified inception module (**A**) and module (**B**).

Based on the above discussion, this paper proposes a structure similar to Inception, as shown in Figure 9, to retain more feature information by multi-scale feature extraction and then fusion. Four convolutional kernels of different sizes, 1×1, 3×3, 5×5 and 7×7, are used to extract features from the same input in parallel, and finally the results of these parallel feature extraction are fused and fed into the next layer of the network. The smaller convolutions can extract local features, and the larger convolutions can learn the global features. The convolution kernels of 5×5 and 7×7 are selected to cover the feature maps of different sizes in YOLOv3 to ensure that the target information on the feature maps is obtained. The 1×1 convolution can reduce the model parameters, and adding 1×1 convolution before 7×7 convolution (with 128 channels) to adjust the number of channels can reduce the parameters by 75%. The maximum pooling operation preserves the maximum value in the region to provide transfer flip invariance, extracting the main features of the image while making the feature map compressed to a smaller size, reducing the complexity of the model feature computation.

**Figure 10:**
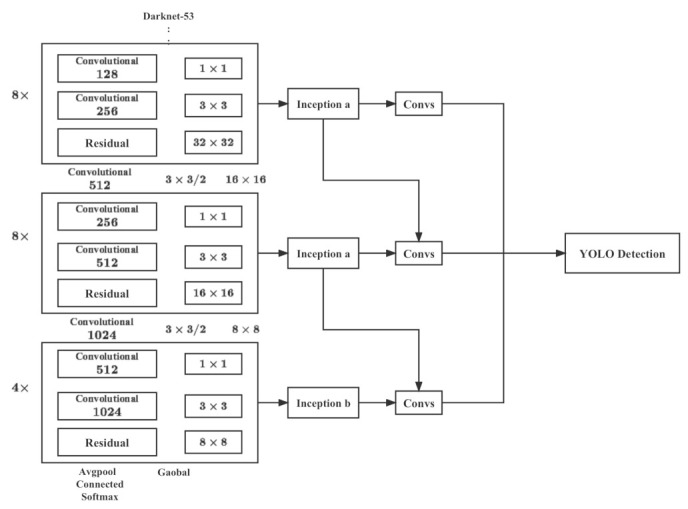
Structure of our inc-YOLO. This model incorporates the original Darknet and Inception structure including Inception A and Inception B in Figure 9.

The structure of inc-YOLO model proposed in this paper is shown in Figure 10, by adding module a and module b in Figure 9 to YOLOv3 before feature fusion, so that the low and high level feature maps are first connected to the Inception module before fusion, and the same layer of feature maps are connected after the multi-scale feature extraction.

The multi-scale feature extraction is performed, and the multi-scale feature fusion results are obtained before the connection is made to obtain richer feature information. The size of the low-level feature map is 52×52×256, which is connected to the a module for feature extraction by four branches with the ratio of 1:1:1:1, while the size of the high-level feature map is 13×13×1024, which retains relatively limited detailed feature information after Darknet multi-layer convolution. The Inception b module performs multiscale feature fusion from three branches, and the ratio of branch channels is 2:1:1. Since the low-level feature map has more fine-grained feature information than the high-level feature map, more diverse features can be obtained through multiscale feature extraction, and more meaningful features can be retained for small objects and occluded parts, which effectively improves the detection of small objects and occluded objects. The effect of the multi-scale feature extraction can be improved. Therefore, the number of channels in the high and low layer fusion retains the ratio of 1:2 in the original YOLOv3 in order to retain more feature information in the low layer feature map and improve the detection accuracy of natural environment including the heavily occluded data set.

### 5.2. Cluster-RCNN

The Faster R-CNN [50] divides the detection process into two parts: the generation of candidate frames that may contain objects and the correction of the candidate frames for classification, which has high detection accuracy and better detection effect for small-scale targets with complex features such as pests and diseases. The RPN network outputs about 2000 proposals containing foreground and background probabilities and border adjustment parameters, while the ROI pooling layer receives both the original feature map and multiple proposals from the RPN network, and unifies the scale output for final target classification and position adjustment. regression adjustment.

Faster R-CNN proposes an RPN network for candidate box selection to significantly reduce the candidate box extraction time by setting *K* initial detection boxes through the Anchor box mechanism, and using a sliding window of 3×3 on the input feature map to generate *K* candidate boxes. The sliding window of 3×3 is used to scan the input feature map, and *K* candidate boxes are generated at each time with the center of the sliding window as the center, and the IoU of the box with the true label ground truth is used to determine whether the box contains the target to be detected, and then the position of the candidate box is adjusted to get the initial candidate box, which makes the candidate box generation speed reach milliseconds.

**Figure 11:**
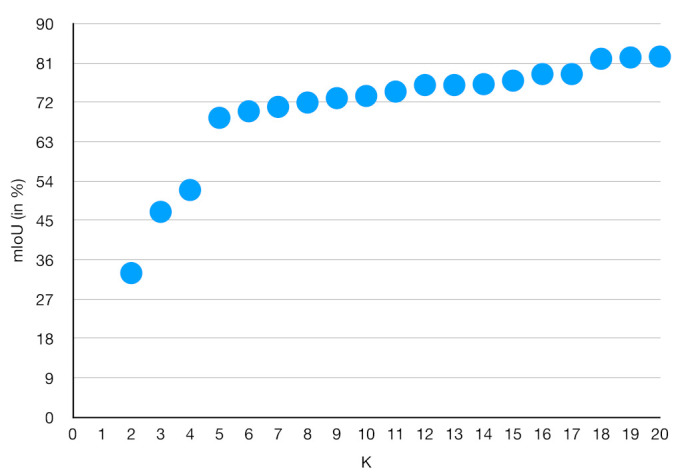
The relationship between the number of clusters *K* and model performance.

The default size of 9 Anchor boxes used in the original Faster R-CNN does not meet the detection requirements of the dataset in this paper. In this paper, we want to propose a network model for detecting dozens of pests and diseases with different feature sizes. Therefore, the *K*-means algorithm is used to cluster the anchor sizes for pest detection, and the distance measure used is:

In this paper, the range of *K* values is [2,20], and the relationship between *K* values and mIoU is obtained by K-means clustering of the pest data set, as shown in Figure 11.

It can be seen that the IOU curve starts to level off when *K* is taken as 18. Therefore, for the characteristics of the pest data set in this paper, a small size anchor box is added to the original size, and the final size and aspect ratio are [64,128,256,512] and $\left[\frac{1}{2},1,2\right]$ respectively, and the RPN network generates 18 anchor boxes of different sizes and proportions according to the center point each time sliding to roughly cover more pests and diseases, so as to improve the detection accuracy of pests and diseases.

### 5.3. Transfer Learning

In order to make the model converge faster and have stronger generalization ability, this paper chooses to use migration learning technique. The selection of a suitable pre-training model is the key to the success of migration learning, and the most important point is that the dataset of the pre-training model has some correlation and similarity with the dataset of this experiment. In this paper, we use the weight files after pre-training on ImageNet, a huge natural image database with more than 15 million images and more than 20,000 categories. Migrating its weights will be of great help to the model training in this experiment.

In the model improvement, fine-tuning strategy is used, the main idea of which is to adjust one or more layers of the pre-trained model to fit the target task. This experiment retains the convolutional layer weights of the ResNet model. This is because the convolutional layer parameters are used to extract Generic features of the image, which are very helpful for the task of this experiment. The specific changes to the other layers are as follows:

1. Fill the image with “0” values in the form of 2×2 around the image before it is input to the model to better extract the image edge information and control the feature map size.
2. Migration of the convolutional weights from the pre-trained model to the convolutional layer of the model, allowing the weights to be updated simultaneously with the training
3. Add 1 average pooling layer after the convolution layer with a pooling window of 2×2. Calculate the average of the image feature matrix 2×2 region, which helps to preserve more detailed information of the image.
4. then uses the Flatten layer to convert the input of the multidimensional matrix into a one-dimensional matrix to speed up the computation.
5. After the flatten layer, there are 2 fully connected layers with 1 batch normlalization layer between them, which can speed up the training and improve the accuracy at the same time. The first fully-connected layer has an output dimension of 1024 and uses ReLU as the activation function.

### 5.4. Experiments Metircs

#### 5.4.1. Precision and Recall

Since some indicators are statistical indicators, let’s review the relevant statistics first. There are two kinds of mistakes we make when doing hypothesis testing:

1. The original hypothesis is correct, and judge it to be wrong.
2. The original hypothesis is wrong, and judge it to be correct.

These two types of errors are the first type of error and the second type of error, respectively, as shown in Table 5.

*TP* denotes the number of samples that are positive but predicted to be positive, *FP* denotes the number of samples that are negative but predicted to be positive, *FN* denotes the number of samples that are positive but predicted to be negative, and *TN* denotes the number of samples that are negative but predicted to be negative.

The *Precision* is the probability of detecting the correct target among all detected targets, as shown in Equation (2).

(2) $$Precision=\frac{TP}{TP+FP}$$

*Precision* is defined in terms of the predicted outcome. Note that Precision and Accuracy are not the same, as accuracy is for all samples, while *Precision* is only for the fraction of samples detected (including false positives).

(3) $$Recall=\frac{TP}{TP+FN}$$

*Recall* is the probability that all positive samples are correctly identified, as shown in Equation (3). *Recall* is from the perspective of the sample. *Recall* is also known as the check-all rate.

#### 5.4.2. Average Precision

Check accuracy and check completeness are contradictory measures; in general, when check accuracy is high, check completeness tends to be low; and when check completeness is high, check accuracy tends to be low. If we want as many good melons to be selected as possible, we can increase the number of melons to be selected. If all the melons are selected, then all the good melons will be selected, but the accuracy rate will be lower. Usually, only in some simple tasks, it is possible to make both the check-all rate and the check-accuracy rate high. Therefore, in order to measure the performance of the model more comprehensively, *AP* is proposed. *AP* represents the average value of the detector in each Recall case, and from a discrete perspective *AP* can be expressed as follows:

(4) $$AP=\frac{\sum P_{r_{i}}}{\sum r}$$

where $P_{r}$ denotes the *P* value corresponding to r−i on the *PR* curve, and ∑r=1. Obviously, AP is specific to a category, and mAP averages AP over the dimensions of the category, as shown in Equation (5).

(5) $$mAP=\frac{\sum_{i=1}^{k}\left(AP_{i}\right)}{k}$$

Thus the performance of the multi-classifier can be evaluated. *mAP* must be of size in the interval [0, 1], the larger the better.

#### 5.4.3. Frames Per Second

*FPS* refers to the number of Frames Per Second, which is a measure of the amount of information used to save and display dynamic video. The more frames per second, the smoother the action displayed will be. In a deep learning model for object detection, *FPS* is used to represent the inference speed of the model.

### 5.5. Platform and Parameters

A personal computer (CPU: Intel(R) i9-10900KF; GPU: NVIDIA RTX 3080 10 GB; Memory: 16 GB; OS: Ubuntu 18.04, 64 bits) is used to carry out the entire model training and validation process. The Adam optimizer with an initial learning rate, $a_{0}$ = 1$e^{-4}$ is selected in this paper.

## 6. Discussion

### 6.1. Analysis of the Effect of Sub-Class Detection

To further improve the detection effect of this model, we show the results of the model for each subclass in this section, as shown in Table 6.

From the above table, we can see that there are several characteristics of the detection accuracy for different subclasses:

1. In most cases, the detection performance increases as the growth stage of the pest changes, with the highest detection accuracy for adults.
2. There are significant differences in the detection performance of different stages, among which the detection accuracy of egg stage is the lowest.
3. The subclasses with low number of training sets generally have low detection results.

### 6.2. Weak Class Detection Improvement

By observing the results of the model for each pest, as discussed in Section 6.1, we can find that the best detection effect is for the *Anoplophora chinensis* (adult) and the worst detection effect is for the *Clostera anachoreta* (egg). The main reasons for this analysis are:

1. The *Anoplophora chinensis* (adult) are more obvious and less affected by the quality of the image.
2. The number of training set of *Clostera anachoreta* (egg) is small, which contains only about 62 images. And the number of number of training set of *Anoplophora chinensis* (adult) is about 758.
3. The object scale of *Clostera anachoreta* (egg) is too small, although the anchor box is improved to improve the detection accuracy, the detection accuracy is still slightly lower than that of other diseases with larger scale.

Based on the above findings, we have made two improvements:

1. Clustering more appropriate anchor box according to the small size of the bounding boxes.
2. Use some dataset enhancement methods for the problem of unbalanced dataset.

In this paper, we test each improvement point according to the principle of variable control experiment, and the comparison test results are shown in Table 7.

The mAP of all testset and *Clostera anachoreta* (egg) before improvement were 85.0% and 69.6%, respectively. When only anchor was improved, the mAP of testset and *Clostera anachoreta* (egg) improved by 0.2% and 4.7% percentage points, respectively. The mAP of small-scale objects improved significantly. When only data augmentation is performed, the mAP of *Clostera anachoreta* (egg) improved by 8.9% percentage points. Thus, all the improvement methods proposed in this paper have improved the mAP of pest detection to different degrees.

### 6.3. Application

**Figure 12:**
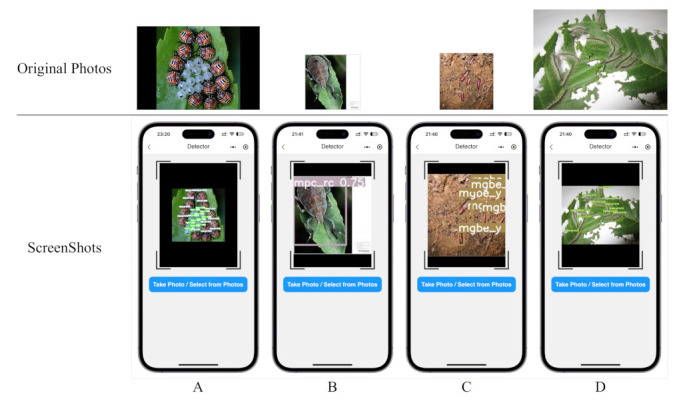
Screenshot of the application detection, top is the original image in different resolutions, bottom is the screenshot of the detection result. (**A**–**D**) shows the detection results for different densities and resolutions of the original image.

In order to apply the proposed method to a real agricultural scenario, we developed an application based on the Android and iOS platform using Wechat development SDK, as shown in Figure 12. We also validated it on the wheat blast dataset.

From the above figure, we can see that even intensive recognition tasks, such as Figure 12A,C,D, can be detected very well on the mobile side. Moreover, the figure shows that even images of different sizes, i.e., different resolutions, can still be detected well, such as Figure 12B,D. The screenshot shows the iPhone 14 Pro detection, and since our application is based on wechat, it can also be run on Android. In order to ensure that the model can run smoothly with mobile, we pruned the model appropriately. The pruned model can reach 13.7 FPS on Huawei Mate 20 Pro, and the mAP can reach 67.4%. This result can already help users to make preliminary judgments on pests and diseases.

### 6.4. Limits and Feature Works

The proposed method improves the pest detection accuracy, but there is still room for improving the average detection accuracy due to the large number of pest species, the small size and variable shape of some pests, or the similarity of features such as color and texture, and the large number of pest images with natural backgrounds that are not of interest to the detection errors. At the same time, by combining the advantages of each model and dividing the detection steps, the proposed method improves the accuracy of the model but also decreases the detection speed. These will be the focus of the next research in this paper.

## 7. Conclusions

Protecting crop yields is the most important aspect of agricultural production, and one of the important measures in preserving yields is the control of crop pests and diseases. Therefore, the identification of crop pests and diseases is of irreplaceable importance. In recent years, with the maturity of computer vision technology, more possibilities have been provided for implementing plant disease detection.

However, although deep learning methods are widely used in various computer vision tasks, there are still limitations and obstacles in practical applications. Traditional deep learning-based algorithms have some drawbacks in this research area: (1) Recognition accuracy and computational speed cannot be combined. (2) Different pest and disease features interfere with each other and reduce the accuracy of pest and disease diagnosis. (3) Most of the existing researches focus on the recognition efficiency and ignore the inference efficiency, which limits the practical production application.

Based on the above problems, in this study, an integrated model integrating single-stage and two-stage target detection networks is proposed. The single-stage network is based on the YOLO network, and its internal structure is optimized; the two-stage network is based on the Faster-RCNN, and the target frame size is first clustered using a clustering algorithm in the candidate frame generation stage to improve the detection of small targets. Afterwards, the two models are integrated to perform the inference task.

For training, we use transfer learning to improve the model training speed. Finally, among the 37 pests and 8 diseases detected, this model achieves 85.2% mAP, which is much higher than other comparative models. After that, we optimize the model for the poor detection categories and verify the generalization performance on open source datasets. In addition, in order to quickly apply this method to real-world scenarios, we developed an application embedded in this model for the mobile platform and put the model into practical agricultural use. The main contributions of this paper include:

1. By adding the Inception module to the YOLOv3 model and using four different sizes of convolutional kernels (1×1, 3×3, 5×5, 7×7) to perform multi-scale feature extraction and fusion in parallel, this paper proposes the inc-YOLO model;
2. by clustering the anchor box and increasing the anchor box types, this paper proposes the cluster-RCNN model, and achieves a better localization effect;
3. By integrating the above two models, our method finally achieves 85.2% accuracy in the detection of 37 pests and 8 diseases. This method provides a feasible solution to achieve fast and efficient pest and disease detection based on mobile platform.

## Figures and Tables

**Table 1:** Comparisons of different detection networks’ performance (in %).

Model	*Input* Size	*Precision*	*Recall*	*mAP*	*FPS*
MobileNet	416×416	58.3	55.2	56.7	34
Faster-RCNN	416×416	60.7	59.3	60.3	7
SSD [43]	300×300	63.8	59.3	58.7	24
RefineDet [44]	300×300	67.8	62.1	65.9	15
EfficientDet [45]	416×416	72.1	69.2	69.7	7
YOLO v3	608×608	79.7	79.4	79.5	28
YOLO v5 [46]	608×608	81.0	78.6	80.7	33
Our Methods					
Inc-YOLO	608×608	79.3	80.8	79.5	26
Cluster-RCNN	416×416	82.7	83.0	82.5	18
Model Ensemble	608×608	85.2	84.8	85.0	23

**Table 2:** Distribution of different species of pest data sets.

Pest	Egg	Larva	Cocoon	Pupa	Adult
*Anoplophora chinensis*	-	276	-	-	758
*Micromelalopha troglodyta Graeser*	-	398	-	-	322
*Apriona germari Hope*	-	371	-	-	385
*Erthesinafullo* (Thunberg)	338	419	-	-	247
*Sericinus montelus Gray*	244	367	-	-	295
*Cnidocampaflavescens* (Walker)	-	472	43	-	392
*Clostera anachoreta*	62	288	-	-	387
*Hyphantria cunea*	264	343	-	276	38
*Psilogramma menephron*	-	34	-	355	459
*Plagiodera versicolora*	501	521	-	-	298
*Parasa consocia*	-	534	-	-	45
*Monochamus alternatus*	-	58	-	-	442
*Drosicha corpulenta* (Kuwana)	-	487	-	-	72
*Spilarctia subcarnea* (Walker)	-	492	-	-	425

**Table 3:** Distribution of disease data sets.

Species	Numbers	Numbers
Peach	378 (bacterial spot)	-
Pepper	896 (bacterial spot)	-
Potato	952 (early blight)	977 (late blight)
Squash	914 (powdery mildew)	-
Strawberry	1253 (leaf scorch)	-
Tomato	965 (curl virus)	864 (mosaic virus)

**Table 4:** Model robustness validation experiments.

Model	Pretrained	*mAP*
MobileNet	COCO	32.8 [47]
MobileNet	COCO + PVD	22.4 [47]
Faster-RCNN	iNaturalist	36.1 [47]
Faster-RCNN	COCO	38.9 [47]
Our Methods		
Inc-YOLO	COCO	47.1
Cluster-RCNN	iNaturalist	52.3
Model Ensemble	-	54.8

**Table 5:** Matrix of classification metrics.

Label/Prediction	Positive	Negative
Positive	TP	FP
Neagtive	FN	TN

**Table 6:** mAP of each subclass in pest dataset.

Pest	Egg	Larva	Cocoon	Pupa	Adult
*Anoplophora chinensis*	-	79.1	-	-	89.6
*Micromelalopha troglodyta Graeser*	-	83.4	-	-	89.4
*Apriona germari Hope*	-	79.9	-	-	87.7
*Erthesinafullo* (Thunberg)	77.1	82.3	-	-	88.9
*Sericinus montelus Gray*	78.3	85.1	-	-	86.4
*Cnidocampaflavescens* (Walker)	-	85.9	85.5	-	85.8
*Clostera anachoreta*	69.6	82.5	-	-	88.2
*Hyphantria cunea*	77.8	83.7	-	86.7	86.1
*Psilogramma menephron*	-	86.2	-	85.9	87.6
*Plagiodera versicolora*	78.5	84.7	-	-	85.4
*Parasa consocia*	-	83.8	-	-	87.3
*Monochamus alternatus*	-	84.2	-	-	89.2
*Drosicha corpulenta* (Kuwana)	-	83.1	-	-	85.1
*Spilarctia subcarnea* (Walker)	-	85.4	-	-	84.5

**Table 7:** mAP of weak subclass and all testset with / without improvement.

Method	*Clostera anachoreta* (Egg)	All Testset
baseline	69.6	85.0
Appropriate anchor	74.3	85.2
Data Augmentation	78.5	85.0
Both	81.4	85.2
